# Second Edition of the Scientists of Tomorrow/*Cientistas do Amanhã* Project: advancing scientific thinking, methodology, and equitable education

**DOI:** 10.31744/einstein_journal/2025AE1513

**Published:** 2025-07-11

**Authors:** Érika Bevilaqua Rangel, Victoria Tomaz, André Luiz Teles e Silva, Érica Kássia de Sousa Vidal, Isabella de Sousa Nóbrega, Alexandre Giannecchini Romagnolo, Olívia Furiama Metropolo Dias, Marcella Liciani Franco, Guilherme Ferreira de Britto Evangelista, Pedro Cancello, Thiago Pinheiro Arrais Aloia, Guilherme Martelossi Cebinelli, Lorrayne Belotti, Letícia Yamawaka de Almeida, Ilana Eshriqui Oliveira, Daiana Bonfim, Raymundo Machado de Azevedo, Ronaldo Hueb Baroni, Marcella Zuliani Lopes Soares, Priscilla Cerullo Hashimoto, Thomaz Bittencourt Couto, João Carlos Barbosa, Luciana Cintra, Camila Hernandes, Elisa Harumi Kozasa, Eliseth Ribeiro Leão, Lionel Fernel Gamarra, Luciano Cesar Pontes de Azevedo, Luiz Vicente Rizzo

**Affiliations:** 1 Stricto Sensu Postgraduate Program in Health Sciences Hospital Israelita Albert Einstein São Paulo SP Brazil Stricto Sensu Postgraduate Program in Health Sciences, Hospital Israelita Albert Einstein, São Paulo, SP, Brazil.; 2 Escola Municipal de Ensino Fundamental Professor Paulo Freire São Paulo SP Brazil Escola Municipal de Ensino Fundamental Professor Paulo Freire, São Paulo, SP, Brazil.; 3 Centro de Estudos, Pesquisas e Práticas em Atenção Primária e Redes Instituto Israelita de Responsabilidade Social Albert Einstein São Paulo SP Brazil Centro de Estudos, Pesquisas e Práticas em Atenção Primária e Redes, Instituto Israelita de Responsabilidade Social Albert Einstein, São Paulo, SP, Brazil.; 4 Hospital Israelita Albert Einstein São Paulo SP Brazil Hospital Israelita Albert Einstein, São Paulo, SP, Brazil.

**Keywords:** Science, Scientific methodology, Elementary school, Soft skills, Quality education, Delivery of health care, Educational personnel, Schools, Students, Brazil

## Abstract

The Scientists of Tomorrow project is a partnership between the Postgraduate Program and multidisciplinary teams of the Hospital Israelita Albert Einstein and Escola Municipal de Ensino Fundamental Professor Paulo Freire of Paraisópolis in São Paulo, Brazil. To inspire 9th-grade students, the project introduced scientific research topics and methodologies to answer questions. This four-month program covered cellular and molecular biology, nanotechnology, bioinformatics, big data, neuroscience, and the impact of nature on well-being and innovation. In the second edition, the program featured 17 students and 35 activities from the beginning of August to the end of November 2023. The program connected with clinical practice, offering students insights into pre-clinical and clinical studies, basic life support training, an overview of the Brazilian Public Health System, and anxiety management techniques such as meditation. Diverse teaching strategies enhanced learning, as evidenced by theory-practical classes, pre- and post-tests (p<0.05), Kahoot! quizzes, design thinking, and real-time feedback. Students were encouraged to express their ideas verbally and in writing, individually and in groups. On the final day, the students presented their work in poster sessions, where they answered questions, reflected on their research, interacted with other researchers, and developed communication skills. As an innovative and replicable initiative, Scientists of Tomorrow empowers students to apply knowledge effectively, integrating scientific and technological skills with core values. Aligned with the United Nations Sustainable Development Goals (SDGs), especially SDGs 4 (quality education) and 10 (reducing inequalities), this project promoted continuous learning and self-improvement, preparing students to navigate real-life challenges with flexibility, resilience, and creativity.

## In Brief

Rangel et al. documented the experience of the second edition of the Scientists of Tomorrow/Cientistas do Amanhã project, a four-month training initiative in health sciences involving 17 students from a public primary school. The training featured hands-on laboratory activities, expanded students’ knowledge at the interface of research and clinical practice, taught them how to formulate scientific questions, and used appropriate methodologies to address them. Emphasizing an immersive, student-centered approach, the project also focused on soft skill development, culminating in a poster session in which students presented their scientific work to postgraduate students and researchers at Hospital Israelita Albert Einstein.

## Highlights

■ Ensuring quality education, providing vocational training, and promoting communication technology, technical, and scientific programs are goals set by the United Nations to enhance learning opportunities for all.

■ Critical thinking, fostered through theoretical and practical activities, is essential for building relevant skills, including literacy and numeracy.

■ The Scientists of Tomorrow/Cientistas do Amanhã project is a reproducible initiative that can be implemented in other research centers and schools.

## Graphical abstract

**Figure:**
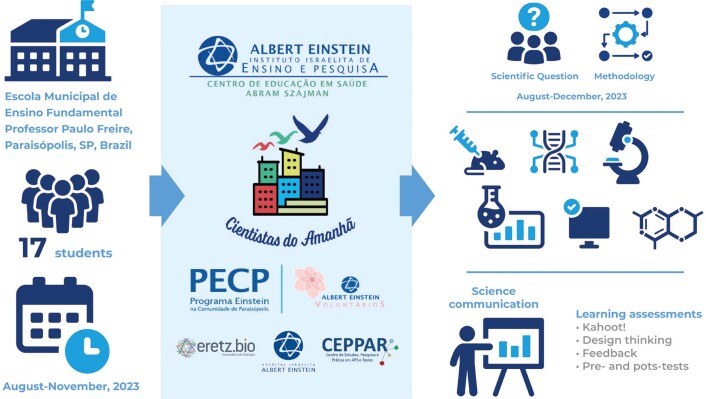

## INTRODUCTION

In 2022, the first edition of the Scientific Initiation project, titled Scientists of Tomorrow/*Cientistas do Amanhã*, was launched at *Hospital Israelita Albert Einstein* (HIAE).^1^ This initiative involved 15 students from *Escola Municipal de Ensino Fundamental Professor Paulo Freire,* which allowed us to critically analyze the teaching of science to these students and establish a foundation for future editions.

Our primary objective was to foster scientific thinking among elementary school students, emphasizing the understanding of scientific methodology through participation in theoretical and practical activities related to scientific research. These activities were conducted under the guidance of faculty and postgraduate students from the *Programa de P*ós-*Graduação Stricto Sensu em Ciências da Saúde* (Stricto Sensu Postgraduate Program in Health Sciences) at the *Instituto Israelita Albert Einstein* students from the medical school at the *Faculdade Israelita de Ciências da Saúde Albert Einstein* (FICSAE), and the multidisciplinary team at HIAE. Additionally, we aimed to demonstrate how scientific research can address everyday problems and serve as a valuable tool for meeting societal needs.

To achieve these objectives, we set the following specific goals:

- make scientific knowledge accessible to public school students and integrate it into their daily lives to help transform their reality,
- strengthen interactions between academic institutions and public elementary schools,
- promote better learning conditions and socialization for young people, fostering their academic progress and social integration, and
- encourage strategies to improve teaching and learning conditions in alignment with local, regional, and global contexts.

The second edition of the project ran from August 1^st^ to November 28^th^, 2023, and featured 35 activities held from 2:00 to 5:00 p.m. on Mondays and Tuesdays. Seventeen ninth-grade students from *Escola Municipal de Ensino Fundamental Professor Paulo Freire* participated in these activities. In this edition, we introduced strategic adjustments to create a more immersive, hands-on learning experience than the first one. Emphasizing a student-centered approach, we encouraged both individual and collaborative growth through dynamic teaching methods. Our efforts focused on fostering scientific thinking, strengthening students’ abilities to communicate scientific concepts verbally and in writing, and enhancing their practical engagement with science.

### Description of the Scientist of Tomorrow/*Cientistas do Amanhã* project: an immersive program

We invited three students from the first edition of the Scientists of Tomorrow/*Cientistas do Amanhã* project—João Gabriel Barbosa da Costa, Luiz Felipe Santana Machado, and Maria Eduarda de Souza Neves Costa—to visit *Escola Municipal de Ensino Fundamental Professor Paulo Freire* and share their experiences, encouraging new students to join. As a result, 35 students participated in short-term immersive training from June 12^th^ to 16^th^, 2023, where they explored topics such as nanotechnology, artificial intelligence, environmental preservation, non-violent communication, innovation, and design thinking to address non-communicable chronic diseases. From these 35 students, 17 were selected to join the long-term program based on evaluations of participation, commitment, and discipline by *Escola Municipal de Ensino Fundamental Professor Paulo Freire* teachers, postgraduate student mentors, and Prof. Érika Rangel.

Below, we outline the 35 activities conducted with the students during their Scientific Initiation training.

### August 2023 (9 activities)

**August 1**^st^**:** Presentation of the Scientific Initiation Project, outlining its benefits and responsibilities, conducted by Prof. Rizzo, a team of postgraduate students, and Prof. Érika Rangel

Prof. Rizzo emphasized the importance of science in public health. He noted that, while we have experienced the COVID-19 pandemic, we must consider the possibility of facing another pandemic in the next 10 years. Such topics are discussed in scientific articles and popular media. He highlighted that science is not solely the domain of scientists; it is also a tool for solving everyday problems. He further highlighted that every problem has a solution, but finding a solution requires asking the correct questions.

The students were highly engaged in the discussion circle, expressing expectations such as “learning more about science,” “opening their minds to the future,” “making the right choices in the future,” and “expanding their knowledge.”

**August 7**^th^**:** Laboratory experiences with postgraduate students Alexandre Giannecchini Romagnolo, André Luiz Teles e Silva, Érica Kássia de Sousa Vidal, Isabella de Sousa Nóbrega, and Marcella Liciani Franco

Seventeen students were divided into two groups for these two activities. In the first activity, students watched videos of viruses, bacteria, and fungi along with basic concepts regarding the characteristics of plant and animal cells. In the second activity, they gained hands-on experience in a research laboratory by engaging in tasks such as using pipettes, learning the importance of proper parameterization, and familiarizing themselves with basic equipment such as centrifuges and precision balances.

After the activities, the students were divided into four groups to participate in an interactive exercise combining theoretical and practical knowledge using the *Kahoot!* tool. This was a moment of great learning and fun, followed by feedback on topics.

**August 8**^th^**:** Peripheral blood mononuclear cell (PBMC) isolation using the Ficoll^®^ method with postgraduate students Victoria Tomaz and Érica

Students participated in an interactive class that explored the various types of cells found in the blood, including red blood cells, platelets, and leukocytes, along with their respective subtypes (neutrophils, basophils, eosinophils, lymphocytes, and monocytes) and their primary functions. They found that eosinophils respond to allergic stimuli and parasitic infections, lymphocytes play a crucial role in combating viruses, and neutrophils are activated to fight bacterial infections. Additionally, the students discovered that decreased levels of red blood cells can lead to anemia, which reduces oxygen transport and results in fatigue.

Following the class, students had the opportunity to observe these cells under a microscope, identifying the morphologies of granular leukocytes (neutrophils, eosinophils, and basophils) and agranular leukocytes (monocytes and lymphocytes) as well as abnormal cells from patients with leukemia.

**Figure 1:**
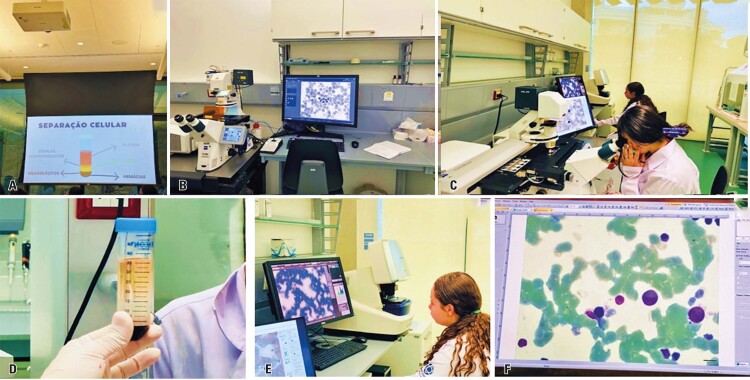
A to F: Hands-on activity using the Ficoll® reagent to separate peripheral blood mononuclear cells

Students then participated in a hands-on activity using the Ficoll^®^ reagent to separate PBMCs, such as lymphocytes and monocytes, through centrifugation. They learned methods for isolating PBMCs and their clinical applications in cell therapy, autoimmune disease diagnosis, and biomarker research. This activity is illustrated in figures 1A-1F.

**August 14**^th^**:** Microscopy activity with the microscopy platform specialist Thiago Pinheiro Arrais Aloia and postgraduate students Marcella and Isabella

On this day, students attended a class on different types of epithelial tissues in the human body. They discovered that epithelial tissues consist of closely packed cells that are tightly bound to one another, lining the body’s outer surface, internal cavities, and organs, while also performing secretory functions. The following classifications were introduced:

- covering epithelium, including i) simple epithelium, comprising a single layer of cells; ii) stratified epithelium, comprising multiple layers of cells; and iii) pseudostratified epithelium, comprising a single layer of cells with varying heights, providing an illusion of stratification,
- classification by cell shape: i) squamous epithelium, composed of flattened cells; ii) cuboidal epithelium, composed of cube-shaped cells; iii) columnar epithelium, composed of elongated, column-shaped cells; and iv) transitional epithelium, composed of cells that are initially cuboidal but eventually become flattened because of organ dilation.

Students observed that the epithelial tissue of the human skin, the body’s largest organ, was stratified, whereas the epithelial tissue covering the organs was simple. They also learned that the glandular epithelium, which shares characteristics with those of the covering epithelium, is found in the exocrine and endocrine glands and serves secretory functions.

The students further discovered that epithelial cells play vital roles beyond lining surfaces and secrete substances. They are involved in absorbing molecules and water, as observed in the kidneys and intestines, and exhibit sensory functions related to smell and taste.

**Figure 2:**
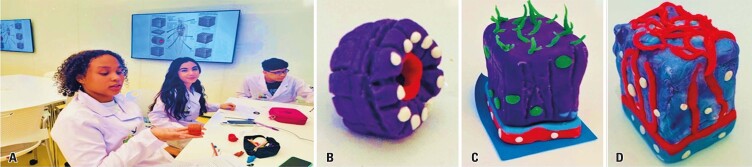
A to D: A class on epithelial types and functions, along with modeling the various types of epithelial tissues

During the practical activity, students scraped the inner epithelial mucosa of their cheeks and prepared slides for microscopic observation. They also used modeling clay to represent different forms of epithelium, including the renal epithelium (student Thomas Henrique Alves Oliveira), nasal epithelium (student Jamiris Souza Neves), and gastric epithelium (student Alessandra Sousa Belarmino). This activity is illustrated in figures 2A-D.

**August 15**^th^**:** Flow cytometry activity

Herein, the students participated in a hands-on flow cytometry activity titled “Discovering the Power of Immune Cells Using Flow Cytometry.” They learned to characterize immune cells by analyzing their morphological parameters using monoclonal antibodies coupled with fluorochromes for staining.

Guilherme Cebinelli, a flow cytometry platform specialist, first explored the students’ prior knowledge of immune cell morphology to determine how morphological parameters can be measured via light scattering in a flow cytometer. They then measured the cell size and complexity from a peripheral blood sample and discussed the data to identify lymphocytes, monocytes, and granulocytes.

In the second part of the activity, the focus shifted to how a flow cytometer excites the different fluorochromes and detects the fluorescence emitted from them. After discussing laser wavelengths, fluorochrome excitation, and fluorescence emission, Guilherme Cebinelli removed the bandpass filters for violet, blue, and red light, allowing students to observe light from specific bands in the spectrum. The students were excited to see firsthand how a flow cytometer “sees” and differentiates between wavelengths of light emitted by the fluorochromes.

Finally, the students analyzed the results of CD3, CD4, and CD8 staining using fluorochrome-coupled monoclonal antibodies. This exercise aimed to introduce them to the diverse immune cell populations and inspired curiosity in the vast world of immunology.

**August 21**^st^**:** Understanding Big Data with Prof. Helder Nakaya and postgraduate student Alexandre

Professor and biologist Prof. Helder Nakaya began his presentation by stating, “You may not realize it, but if you have ever participated in biological research in a laboratory, you have likely engaged in integrative biology.” He explains that integrative biology involves combining biologically relevant data from multiple sources. For instance, when studying a group of individuals with a viral infection, researchers measure the levels of cytokines and C-reactive protein in real-time to detect viral DNA and assess the correlations between these measurements.

Expanding on this idea, Prof. Helder described how big data is integrated on a larger scale by incorporating vast datasets and more sophisticated processing techniques. In addition to cytokines and PCR, this integration process includes measuring the expression of 50,000 human genes under various conditions in different individuals. This comprehensive approach involves the collective analysis of clinical, immunological, demographic, and symptomatic data to create a complex network of information that leads to new discoveries.

Prof. Helder also highlighted the practical applications of this approach in clinical settings, particularly for understanding the complexity and heterogeneity of diseases, such as COVID-19. Through integrative biology, researchers can stratify patients according to their risk, optimize resource allocation, and predict future pandemics. These pioneering methods lay the foundation for analyzing large epidemiological databases, identifying disease transmission hotspots, integrating transcriptome data with clinical and immunological information, and using machine learning to interpret and analyze microscopic images.

**August 22**^nd^: Introduction to Bioinformatics by postgraduate student Victoria

Victoria began the class by explaining the critical role of bioinformatics in analyzing molecular biology concepts and emphasizing the importance of defining codes. They posed questions such as “How many genes do we have?” “How many of them are transcribed?” “What differentiates DNA from RNA?” and “Can viruses be composed of RNA or DNA?” After engaging the students and clarifying these fundamental concepts, they emphasized that bioinformatics programming makes it possible to predict viral sequences once the algorithms are validated.

During the practical session, each student worked using individual computers and was tasked with identifying the SARS-CoV-2 variant present in an infected individual using programming techniques, including Python. They started by performing quality control of the viral genome sequence, mapping it, and identifying the sequence corresponding to a specific variant. Students carefully documented information about the variant and constructed a potential genome that could predict it, understanding that a viral variant represents a genome with one or more mutations.

Each student then identified the SARS-CoV-2 variant that infected the patient, such as the Omicron variant, delineating its mutations (N501Y, D614G, K417N, and T478K).

**August 28**^th^**:** Visit to the imaging section of the HIAE with Dr. Ronaldo Baroni, Dr. Adham Castro, and postgraduate students Alexandre, Isabella, and Marcella

Dr. Baroni provided an insightful overview of the evolution of radiological examination. He discussed the advantages and importance of X-rays, followed by an explanation of the principles of ultrasound, comparing it with the echolocation used by bats and sonar in submarines. He then introduced the revolutionary impact of computed tomography (CT), pioneered by Godfrey Hounsfield, highlighting its exceptional image resolution, which is further enhanced using iodine-based contrasts, and its ability to scan multiple areas of the body within a short period. He traced the progression of CT from its initial use in skull imaging in the 1970s, the development of helical CT in the 1990s and the advent of multidetector CT with 16 or 64 channels, and more recent versions equipped with dual tubes and 320 channels. He also addressed the importance of monitoring and reducing radiation exposure during CT examinations.

Dr. Baroni also described magnetic resonance imaging (MRI) and highlighted its superior contrast resolution, which is enhanced using gadolinium-based contrast agents. The students learned that an MRI generates a magnetic field 15,000 times stronger than the Earth’s magnetic field. They also discovered that MRI images are produced based on differences in tissue composition and could be captured in any plane. Additionally, they learned that the HIAE was responsible for the first available MRI in Brazil and Latin America in 1986.

Further, Dr. Baroni discussed the pivotal role of artificial intelligence (AI) in advancing radiology studies. He outlined AI applications, such as expediting scans by assessing image quality for accurate diagnoses, prioritizing reports based on severity, developing automatic detection algorithms, integrating imaging with big data for insights, and providing advanced diagnostic and prognostic tools. He cited a specific example of software developed by the HIAE that improved the precision of prostate cancer detection.

In conclusion, Dr. Baroni emphasized the importance of interdisciplinary collaboration between radiologists and other clinical and surgical specialties to enhance patient care. After the presentation, the students toured the HIAE’s image reporting rooms, where they observed X-ray, CT, and MRI procedures, including scans of the abdomen, spine, and heart.

Finally, Dr. Adham Castro discussed the vast opportunities for scientific research on medical imaging. He highlighted the importance of scientific methodology and how innovations in the field are accessible to the global medical community.

**August 29**^th^**:** Anatomy activity, including an explanation of organ functions and a visit to the Anatomy Laboratory with medical students Rômulo Gonçalves Leão and Luana Perrone Camilo from the Scientific Directory Medicine FICSAE and postgraduate students Érica and Alexandre

This class began with an overview of the functions of the heart, lungs, and liver. In the heart segment, fourth-year medical student Rômulo covered key topics such as systole and diastole, the difference between veins (which return blood to the heart) and arteries (which pump blood from the heart), and the structure of the heart, which includes two atria and two ventricles. He explained how the heart receives nutrients and oxygen through the coronary arteries and discussed how conditions such as diabetes and hypertension, as well as lifestyle factors such as smoking and inactivity, can lead to arterial blockage, potentially causing an infarct. Luana, a fifth-year medical student, presented microscopic images of the heart and identified the various cell types present.

Rômulo then shifted focus to the respiratory tract, covering topics on the nose, pharynx, larynx, trachea, bronchi, bronchioles, and alveoli. He highlighted the negative effects of smoking on respiratory cells, particularly on the alveoli, which are crucial for gas exchange in the blood.

Luana then discussed the functions of the liver, including energy storage (glycogen and triglycerides), vitamin A storage, gluconeogenesis, protein production, and drug metabolism. She emphasized how alcohol consumption could damage the liver and impair its function.

For the practical component, students visited the FICSAE anatomy laboratory, where they observed organs from both healthy and diseased deceased donors. They examined an enlarged heart affected by Chagas disease, a heart with infarction, lungs with smoking-related black spots, and cirrhotic livers associated with alcohol consumption. Additionally, the students used microscopy to study the structures of these organs.

### September 2023 (8 activities)

**September 4**^th^**:** Activity at the *Centro de Estudos Pesquisa e Prática em Atenção Primária à Saúde e Redes* (CEPPAR) with Lorrayne Belotti, Letícia Yamawaka de Almeida, Ilana Eshriqui Oliveira, and Daiana Bonfim as well as the postgraduate students Guilherme Ferreira de Britto Evangelista and Isabella

**Figure 3:**
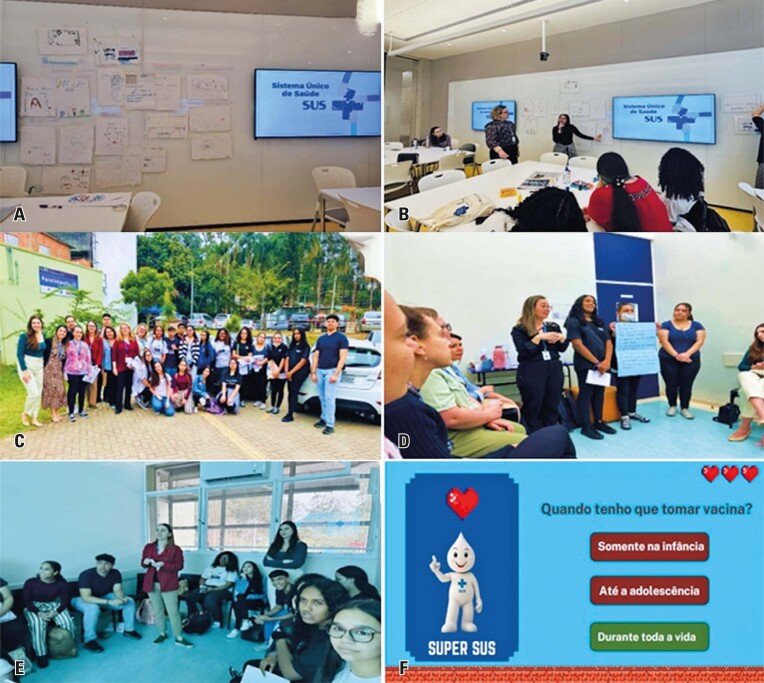
A to F: Leaning about the importance of the SUS (*Sistema Único de Saúde* or Public Health System) in providing equity in healthcare access

Daiana, the coordinator at CEPPAR, began the activity by asking the students, “How do you perceive the SUS (*Sistema Único de Saúde*)?” The students then took 15 min to draw their perceptions. The drawings were displayed on a board, and each student selected another drawing to comment on, as if they were in a museum observing a masterpiece (Figure 3A). The drawings were then categorized into two columns, titled “Challenges” and “Advances,” which facilitated a productive discussion on the positive and negative aspects of the SUS.

Next, the students watched a video titled “*O dia em que o SUS visitou o cidadão*/The day the SUS visited a citizen” (https://bvsms.saude.gov.br/bvs/publicacoes/cordel2.pdf). The video highlighted that the SUS, which was created in 1988, is Brazilian Public Health System (SUS - *Sistema Único de Saúde*), that provides comprehensive care to all Brazilian citizens. It includes various levels of care (primary, secondary, and tertiary) such as Basic Health Units/*Unidades Básicas de Saúde* (UBS), Psychosocial Care Centers*/Centro de Atenção Psicossocial* (CAPS), hospitals, specialty centers, and urgent and emergency services (Figure 3B). The students learned that the SUS was responsible for the vaccination program, sanitary surveillance, and epidemiological surveillance. It is guided by three principles: universality, comprehensiveness, and equity.

**September 5**^th^**:** Activity at the CEPPAR with the previous team and the postgraduate student Victoria: Visiting a UBS and CAPS

Students had the opportunity to visit a UBS and a CAPS in Paraisópolis, the neighborhood where they lived (Figure 3C). They were then divided into two groups: those visiting a CAPS and those visiting a UBS.

During the CAPS visit, the students were engaged in two hypothetical scenarios involving individuals seeking mental health support. They learned how a CAPS is equipped to receive, treat, and rehabilitate these patients, offering group activities, social programs (such as “*Acolhida Noturna*” and *Projeto Terapêutico Singular*”) and art-based therapy, among others.

The UBS group was further subdivided into three groups. The first subgroup visited the oral health team, community health workers (CHW), and nutritionists. They learned about the oral healthcare services provided at the unit, the role of the CHW in registering and monitoring families, and how nutritionists supported individuals with weight issues or other specific needs. The second subgroup visited a nurse, a vaccination room, and another CHW to discuss topics such as suspected pregnancy, the start of prenatal care, rapid tests for syphilis and HIV, and review vaccination cards for adolescents and pregnant women. The third subgroup visited the reception area, a physician, and a CHW, exploring patient flow at the reception, the physician’s role in addressing mental health issues, and how care is provided for adolescents.

After the visits, the groups reconvened to present the hypothetical case they had worked on, discussing the reasons for seeking care at a UBS or CAPS, diagnoses, and strategic plans implemented in the units to improve physical and mental health (Figure 3D). The students received feedback from Diana and her team, which helped deepen their understanding of the SUS and explained their mental health (Figure 3E). She also highlighted the creation of the “*Chega Junto*” program to encourage the participation of adolescents at CEPPAR.

In the final part of the activity, the students participated in a game called “SUPER SUS,” which was designed to reinforce their knowledge of the SUS system (Figure 3F).

**September 11**^th^**:** Visit to the Blood Bank and Cellular Therapy Laboratory with Letícia Taba, Kelen Cristina Alvarez, and Dra. Andrea Kondo and the postgraduate students Victoria and Érica

Letícia discussed blood donation, including an interview covering clinical parameters that concluded with the question “Is my blood safe for donation?” She emphasized the importance of awareness when deciding to donate blood. The students visited the blood bank, where they learned which blood components could be donated, how they were stored, how long they could be stored, and the basic concepts of blood donation/reception based on blood cell type and Rh factor. Afterward, the students visited the cryogenic room with Kelen, who explained cell therapy, including the basic aspects of stem cell research and the use of umbilical cord blood.

**September 12**^th^**:** Blood Typing with postgraduate students Victoria and Érica

On this day, the students were introduced to different types of blood cells (red cells, platelets, and leukocytes), followed by an explanation of the four blood types based on the ABO system (A, B, AB, and O) and the Rh factor (positive and negative). They also learned the importance of compatibility in blood donation and the potential dangers of receiving incompatible blood, which can lead to severe reactions, including hemolysis, shock, and even death.

**Figure 4:**
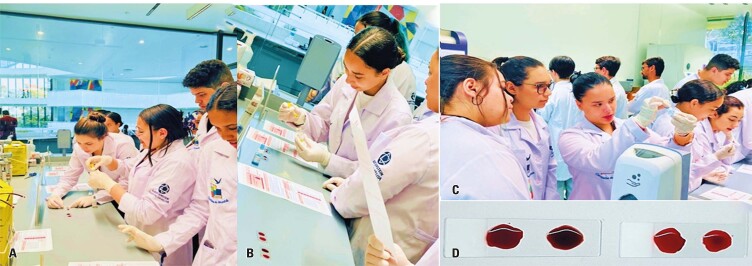
A to D: Learning how to perform blood typing and the importance of this method in clinical practice

During the practical activity, students were divided into eight groups, with each group receiving a blood sample and the necessary reagents to perform an agglutination test and determine the patient’s blood type (Figures 4A-D). Simultaneously, they completed a questionnaire to assess whether ABO and Rh blood types could be safely received. This task was particularly challenging for students because their decisions could directly affect patient outcomes. After the activity, they received feedback that allowed them to connect their theoretical knowledge with practical experience.

**September 18**^th^**:** Discussion of the primary concepts of cell structure, focusing on cytoplasmic membrane transport via osmosis, with research assistant Thiago Aloia and postgraduate student André

In the theoretical class, students reviewed the key concepts of cell structure, including the nucleus, the cytoplasm with its main organelles, and the cytoplasmic membrane. They learned that there are two types of transport across the cell membrane: active transport (phagocytosis, pinocytosis, and exocytosis), which requires energy (ATP), and passive transport (simple diffusion, facilitated diffusion, and osmosis). The importance of these transport mechanisms in maintaining cellular homeostasis was emphasized. Thiago discussed the movement of water across the cell membrane in response to different NaCl concentrations in the extracellular space and introduced the concepts of hypotonic, isotonic, and hypertonic environments.^2^

**Figure 5:**
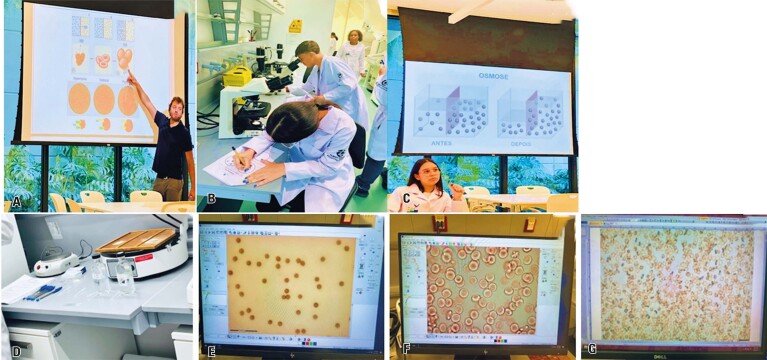
A to G: Theoretical and practical classes on cell membrane transport, including an activity investigating the effect of different concentrations of sodium chloride on red blood cells

During the practical activity, the students observed the red blood cells under a microscope after incubation with various NaCl concentrations (0.4%, 0.6%, 0.9%, and 2.0%). They completed a questionnaire in which they described the changes in the cells and drew the appearance of red cells for each condition (Figures 5A-G). For instance, when incubated with a low NaCl concentration (0.4%), water enters the cells, causing them to swell. In contrast, shrinkage occurred when the cells were placed in a hypertonic solution (2.0% NaCl). At the conclusion of the activity, students participated in *Kahoot!* activities to consolidate their knowledge.

**September 19**^th^**:** Introducing Neuroscience with researchers from the Brain Institute of HIAE: Raymundo Machado, Paulo Bazán, Vanessa Gil Salvatierra, and postgraduate student André

**Figure 6:**
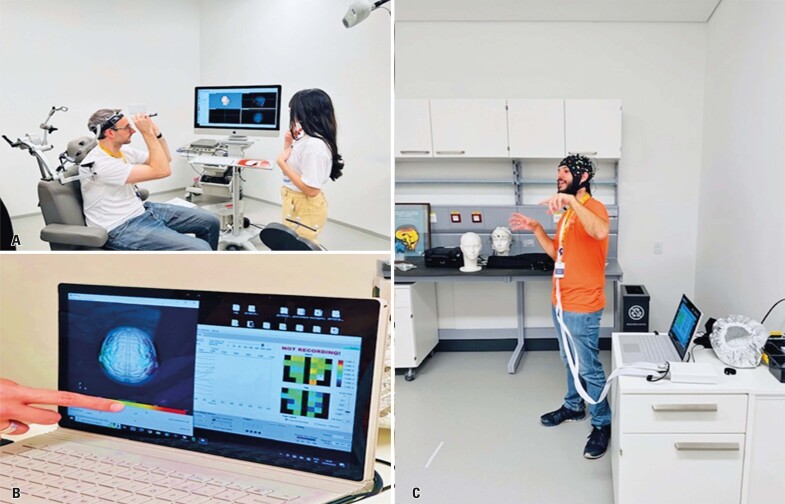
A to C: Discussion on the clinical applications of studies in neuroscience

The Brain Institute’s team organized activities to demonstrate how to investigate brain functions. In the first part of the class, the students were guided to construct the main components of the nervous system. They then discussed neurons, membrane potentials, and action potentials, explaining how these cellular mechanisms lead to physical changes that can be measured using noninvasive techniques such as electroencephalography (EEG), functional near-infrared spectroscopy (fNIRS), and functional MRI (fMRI) (Figures 6A-B).

In the second part of the activity, the students visited the laboratory facilities, where they observed how neurological measurements were made. The team also presented some of their research results, particularly fMRI images.

Additionally, the students had the opportunity to observe and identify brain regions in real-time using infrared cameras and markers. These markers, paired with structural brain images, were used to precisely target brain regions during transcranial magnetic stimulation.

**September 25**^th^**:** Discussion circle on research and science topics with Professor Rizzo and postgraduate students Alexandre and André

Students participated in a relaxed conversation circle with Prof. Rizzo, who began by asking, “What is science?” He then explained that the word “science” came from the Greek word “scientia,” meaning “knowledge.” He then asked, “What is research?” Student Pablo Santos da Silva responded, “It is to find or discover an answer.” Prof. Rizzo added that the term “research” came from the Latin word “perquirere,” meaning “to seek with vigor, with strength.” Next, he asked, “What is scientific research?” emphasizing that it involves a structured investigation.

**Figure 7:**
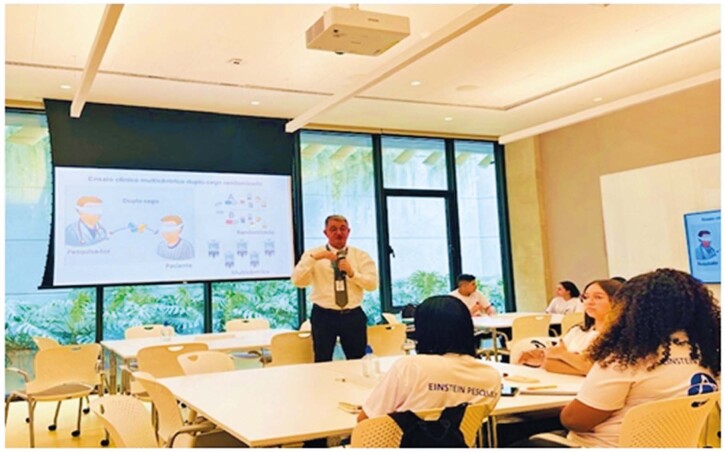
Class about clinical studies with Dr. Rizzo

Prof. Rizzo further explained that research is based on a methodological approach, including observation, questioning, hypothesis formation, experimentation, results, and conclusions. He provided several examples focusing on the key aspects of clinical studies in the field of vaccination, such as the importance of a placebo group for comparison with an intervention group, the process of randomization, and conducting these studies using a multicentric approach to account for human variability. He stressed that understanding these principles is crucial for combating misinformation and fake news and generating clear and reliable information (Figure 7).

**September 26**^th^**:** Anxiety and meditation with Prof. Elisa and Guilherme Romano and postgraduate students André Luiz, Marcella, and Victoria

In this activity, they discussed the theory of anxiety, distinguishing when anxiety can be considered “normal” from when it may be classified as a mental disorder that may require attention from a mental health professional. They also shared strategies with the students to help them better understand and manage their emotions through self-care techniques such as relaxation and meditation.^3^

**Figure 8:**
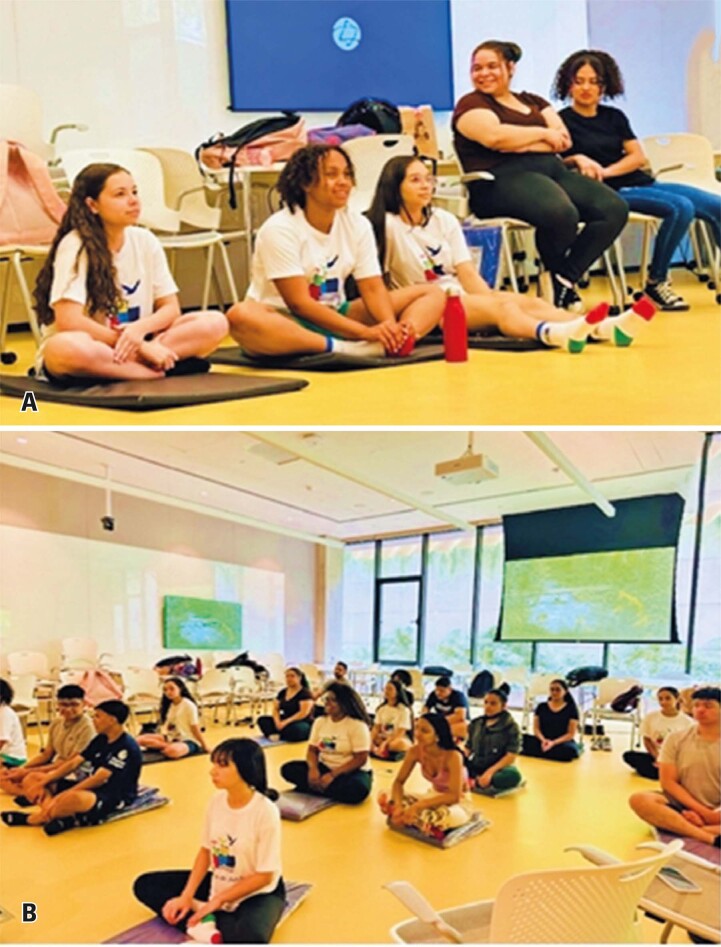
A to B: Practice of relaxation and meditation

The students actively participated in class, acknowledging that while they sometimes sought support from friends, they also recognized the importance of consulting a psychologist during difficult times. Some students had already received psychiatric treatment and understood its significance. They realized the importance of identifying their emotions to improve communication with friends and parents, and enjoyed practicing relaxation techniques and short meditation exercises (Figures 8A-B).

### October 2023 (11 activities)

**October 2**^nd^ **and 3**^rd^**:** Nanotechnology research with Prof. Lionel and his team and postgraduate students Olívia and Victoria

In the first class (October 2^nd^), the students engaged in a practical activity that involved reviewing the sizes and diameters of various particles and cells. They also explored those observable only through an electron microscope (ranging from 0.1 nm to 100 μm, including DNA at ~2 nm, antibodies at 10 nm, and viruses at 90-100 nm), those visible through a light microscope (from 1 μm to 1 mm, including bacteria and mitochondria at 1-3 μm, red blood cells at 7-8 μm, chromosomes at 1-10 μm, and plant and animal cells, which range in size from 10-100 μm), and those visible to the naked eye or with a magnifying glass (larger than 100 μm, including human hair at 60-140 μm, human egg cells at 100 μm, and frog eggs at 1 mm). Notably, they learned that nanoparticle diameters, at 1-100 nm, are much smaller than those of cells (10-100 μm).

Next, with the assistance of monitors from the Nanotechnology team, the students practiced calculating the area, volume, total mass, and density (mass/volume) using small cubes with sides 2 cm long and a large cube with a side 6 cm long. They applied their knowledge to the principles of nanoparticle usage, particularly the increased surface-area-to-volume ratio, which makes nanoparticles effective for delivering drugs to specific cells or tissues, thereby improving treatment efficacy (such as in chemotherapy) while minimizing side effects. Additionally, these drugs have been used as nanoparticles exhibiting fluorescence or enhanced conductivity, making them useful in electronics and imaging applications.

The discussion then shifted to the key applications of nanotechnology in health science, including the research involved in testing new drugs. Various platforms, such as 2D and 3D cell cultures, animal models, and organ-on-a-chip models, were discussed. These platforms were used to assess the toxicity and efficacy of the new compounds. The Nanotechnology team also highlighted that out of 10,000 new compounds, fewer than 250 are tested in pre-clinical research, and fewer than five proceed to clinical trials. During clinical trials, phase 1 studies typically involve 20-100 healthy individuals to assess drug safety; phase 2 studies involve 100-500 patients to evaluate both safety and efficacy; and phase 3 trials include 1,000-10,000 patients to further verify efficacy and monitor adverse events.

In the final part of the class, the students applied the concepts they had learned by selecting the most appropriate platform for testing various products: a new cosmetic (2D or 3D cell culture or organ-on-a-chip), a new drug for Alzheimer’s disease that requires adverse event evaluation (animals), and a new cancer drug to be tested on liver cancer cells (2D cell culture).

The following day, on October 3^rd^, the students worked with polydimethylsiloxane (PDMS) chips, injecting colorful ink to observe the microchannels inside. They also participated in an activity in which breast cancer cells were trypsinized and counted under a microscope, followed by the addition of iron-labeled nanoparticles. The cells were then stained with Prussian blue, and under a microscope, the students observed blue staining inside the cells, indicating that the nanoparticles had entered the cells.

**Figure 9:**
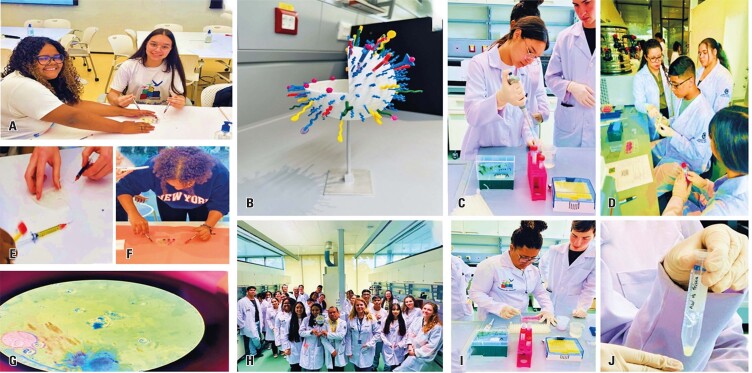
A to J: Practical activity with chips and nanoparticles stained with Prussian Blue inside cells

Figures 9A-J illustrate the nanotechnology-related activities undertaken by the students.

**October 9**^th^**:** Basic Life Support (BLS) Training with nurse João Carlos Barbosa and team and postgraduate student Alexandre

On this day, the students visited the *Centro de Simulação Realística* (CSR)/Realistic Simulation Center and the *Unidade Móvel Einstein* (UME)/Einstein Mobile Unit to learn the fundamental principles of BLS.

Emergency nurse João Carlos led the training, covering essential concepts for the immediate and provisional treatment of trauma or illness victims. This training included wound care, hemorrhage control, fracture immobilization, burn treatment, and management of cardiopulmonary arrest (CPA). The students learned how to identify a CPA, perform cardiopulmonary resuscitation (CPR) effectively, and use an automated external defibrillator (AED).

Under the instructor’s guidance, the students practiced bandaging and bleeding control techniques. They also used high-fidelity simulators to perform CPR and recreate an actual emergency scenario. At the end of the activities, the emergency team was debriefed and provided feedback on key aspects the students need to keep learning.^4^

**Figure 10:**
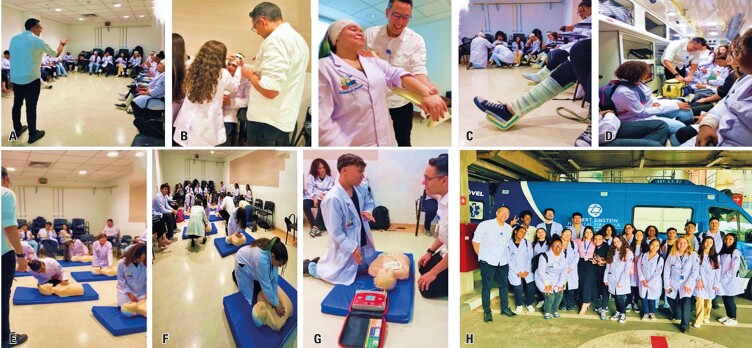
Basic life support training

Following the training, the students visited the UME, where they had the opportunity to explore the ambulance and emergency care infrastructure. They were able to ask questions and became familiar with both essential and advanced equipment. Figures 10A-H illustrate these activities.

**October 10**^th^**and 11**^th^**:** Presentation of the *e-Natureza*/e-Nature program by Prof. Eliseth and her team (Roberta Savieto, Letícia Bernardes, and Sabrina Bomfim)

On the first day of the activity, students were introduced to the *e-Natureza*/e-Nature program, a research initiative that focused on the connection between nature, health, and well-being. They also learned about the e-Nature Ambassadors program, which was launched during the first edition of the Scientists of Tomorrow/*Cientistas do Amanhã* project in 2022.^1^ This program was dedicated to promoting the importance of connecting with and caring for nature. Prof. Eliseth and her team discussed climate change, the significance of connecting with nature, the use of social networks to foster this connection, and the role of empathy in our relationship with nature, including our connection to animals. The students also watched videos highlighting the works of Greta Thunberg, Ryan’s Well Foundation, and William Kamkwamba, which addressed topics such as climate change, the construction of thousands of wells to provide clean water in Africa, and the creation of windmills using bicycle parts and scrap metal to generate electricity in Malawi, respectively.

On October 11^th^, guided by biologist Luciano Lima, a member of the Research Group “e-Nature: Interdisciplinary Studies on the Connection between Nature, Health, and Well-being,” the participants were introduced to various animal and plant species in Burle Marx Park, focusing on the relationship between health and nature. This activity also provided the opportunity to reflect on the theoretical session held the previous day.

**Figure 11:**
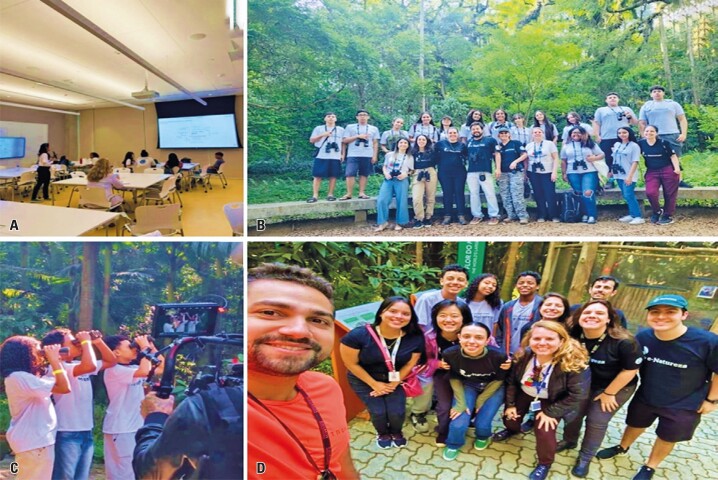
A to D: Class on the importance of nature conservation for human health and well-being, and visits to Burle Marx Park and the *Instituto Butantan*

In 2023, two key activities involved participants from the first edition (2022) of the *Tomorrow’s Scientists* project. In July, three students participated in a data collection activity at the *Instituto Butantan* as part of the research project, *A Time with e-Nature,* gaining hands-on experience in field data collection. Additionally, two students contributed to writing a chapter titled “The Human-Nature Relationship and Social Networks” for the book Nature, Climate, and Public Health*,* which was published in August 2024. These activities are shown in figures 11A-D.

**October 16**^th^**:** Laboratory Exams with Dr. André Mario Doi and Dr. Maira Marranghello Maluf

Drs. André and Maira discussed the definition of microbiology (micro = small and biology), which encompasses the study of microscopic organisms, including bacteria, viruses, archaea, fungi, and protozoans. They reviewed the key concepts of eukaryotes and prokaryotes and explained that prokaryotes are unicellular organisms that lack membrane-bound structures such as the nucleus and other organelles. They also emphasized that not all bacteria and fungi are pathogenic, and that many live in harmony with our bodies. However, when this balance is disrupted, these organisms become pathogenic. They stressed that some bacteria, such as those that cause tuberculosis, tetanus, and cholera, are always pathogenic.

They also explained the discovery of penicillin by the English physician Alexander Fleming in 1928. While studying *Staphylococcus aureus*, a bacterium responsible for wound infections in soldiers, Fleming observed that a substance produced by a fungus of the genus *Penicillium* inhibited the growth of the bacterium. Dr. André highlighted this as an example of the importance of observations in research.

The students also learned how to grow and identify bacteria responsible for diseases using Petri dishes containing different types of agar: blood agar (the most commonly used), chocolate agar (for growing *Haemophilus* and *Neisseria*), and chromogenic agar (which contains specific nutrients and dyes for identifying and quantifying bacteria). Drs. André and Maira then described various methods for collecting bacterial samples, including those from the blood, urine, cerebrospinal fluid, feces, and swabs.

The students then observed the bacterial morphology under a microscope, examining characteristics such as size, shape, arrangement, and structure. They identified different forms, including cocci (round), bacilli (rod-shaped), and vibrio (wavy); arrangements, such as clusters (*Staphylococcus*) and chains (*Streptococcus*); and structures using Gram staining (with crystal violet, Lugol’s iodine, alcohol, water, and fuchsin). The students also learned that Gram-positive bacteria stained blue, whereas gram-negative bacteria stained purple. Moreover, correctly identifying these characteristics helps physicians choose the appropriate antibiotics for infections. In the final part of the class, the students observed parasites preserved in formalin, such as *Ancylostoma*, *Taenia*, and ticks.

**October 17**^th^**:** Visit to the Experimental and Surgical Training Center (CETEC - *Centro de Experimentação e Treinamento em Cirurgia*) with veterinarian Dr. Luciana Cintra

Dr. Luciana explained that small (mice, rats, rabbits, and fish) and large animals (pigs and sheep) could be used in research and innovation projects. However, before animal research can commence, some preliminary steps, including research conducted on cells, must be performed. She also discussed the role of the Ethics Committee, which regulates experiments involving animals, and stated that it is necessary to have a plan before starting these experiments. Specifically, it is essential to have a clear plan before starting experiments, including a well-defined hypothesis, statistical analysis to determine the number of animals required, and adherence to humane criteria for euthanasia.

Dr. Luciana also stressed that animals are no longer used for cosmetic testing and that such analyses are now being conducted using cell cultures and organ-on-a-chip experiments.

**October 23**^rd^**:** Conversation circle on pregnancy, transmissible infectious diseases, and sexuality with Dr. Andrea Hercowitz and Dr. Miguel Cendoroglo

Dr. Hercowitz explained that unplanned pregnancies among teenagers are often the result of insufficient communication within families and misinformation, which can lead to school absenteeism and the discontinuation of education. She emphasized the importance of receiving the human papillomavirus vaccine during adolescence, using barrier methods to prevent sexually transmissible infectious diseases, and the availability of oral or implantable contraceptives. She also discussed HIV transmission and the use of pre- and post-exposure prophylactic measures.

In the final part of the conversation, the Drs. Hercowitz and Cendoroglo addressed transsexuality, the available support systems associated with gender reassignment procedures, and the daily challenges faced by transgender individuals.

**October 24**^th^**:** Design thinking on *diabetes mellitus* (DM) and diabetic kidney disease with Prof. Érika Rangel and postgraduate students Marcella and Alexandre

**Figure 12:**
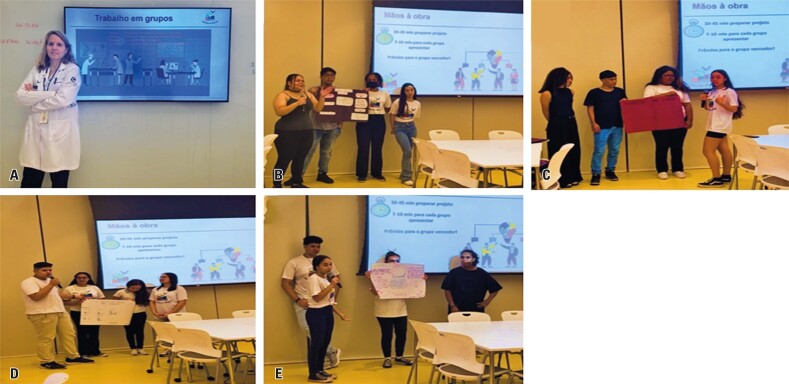
A to E: Design thinking in *diabetes mellitus* research with Prof. Érika Rangel

On this day, the students attended a class on the prevalence of DM, the primary types of DM, and their major micro- and macrovascular complications, including retinopathy, neuropathy, cardiovascular and cerebrovascular diseases, and kidney diseases. They also learned about the modifiable and non-modifiable risk factors for these complications. Using a design thinking approach, the students were challenged to develop four hypothetical projects in groups on the theme of DM: a pre-clinical project, a clinical project involving drug therapy, a clinical project using stem cell therapy, and an epidemiological study. At the end of the activity, they presented their projects to their peers using a poster board with Post-It notes, collated images, and drawings. Notably, all students were actively engaged in the presentations, collaborating as a team and effectively communicating their data in a scientific manner (Figures 12A-E). The projects were structured as posters for the final presentation on November 28^th^.

**October 30**^th^**:** Construction and development of applications with chemical engineer Paulina Achurra from INSPER (*Instituto de Ensino e Pesquisa*)

In this part of the program, the students were again challenged using the design thinking methodology. They were divided into four groups to address the following topics: difficulties in scheduling medical appointments, challenges in booking appointments with specialists, delays in ambulance response times, and lack of access to free medication for the population. The process began with the students interviewing members of other groups, defining problems, brainstorming potential solutions, and working on prototyping applications. At the end of the activity, each group presented the prototype and received feedback from Prof. Achurra.

**October 31**^st^**:** Activity on DNA extraction in the experimental laboratory with Prof. Rizzo and postgraduate students André and Érica

In this activity, the postgraduate students reviewed the structure of DNA, focusing on the types of microorganisms, blood cells, the genetic code, cell structures, nanotechnology, and parasites. They also introduced the concepts of genetic mutations and chromosomal abnormalities. Using a fun hands-on approach, they employed balloons (to represent plasma and cell membranes) and 23 pairs of twines (to represent chromosomes), followed by a competition using *Kahoot!*

Prof. Rizzo then challenged the students by explaining the importance of accessing DNA: (a) it helps us understand our health; (b) when DNA is sequenced, it can predict how many patients may develop a disease; (c) it provides insights into ancestry; (d) it is essential for paternity tests; (e) genetic editing can be used to treat diseases such as sickle cell disease and epidermolysis bullosa; (f) it supports genetic counselling; and (g) it aids in crime investigations, among other applications.

### November 2023 (8 activities)

**November 6**^th^**and 7**^th^**:** Innovation with Vivian Brito, Tauan de Sousa, Bruna Cestari de Azevedo, and Luiz Guilherme Gonçalves Hampariam from Eretz Bio and postgraduate students André and Alexandre

In this activity, students learned about the concept of innovation, which refers to the improvement of products, services, or processes that create value and impact companies, consumers, and society by transforming their behaviors and consumption habits. They also explored four categories of innovation (incremental, radical, disruptive, and open) and examined current examples.

Vivian explained innovation trends in the healthcare market, including improvements in the quality of life, access to healthcare services, disease prevention and early diagnosis, patient empowerment, health education, and responses to emerging challenges. She also discussed Eretz Bio, Einstein’s start-up accelerator, which currently supports 42 start-ups. Vivian briefly described the journey of a start-up and the main challenges it faces. For example, she highlighted a start-up called “We Care - Care Beyond the Skin,” which developed products to mitigate skin and mucosal damage caused by chemotherapy and radiotherapy.

The students were then divided into groups to develop innovative projects in four key areas: ovarian cancer, severe asthma, type 2 DM, and Duchenne muscular dystrophy. They discussed the answers to the questions “What is the disease?” and “What are the main challenges during the patient journey?”, covering various aspects such as diagnosis, treatment, and follow-up. They also researched epidemiological data and prepared presentations. While working in groups, the students practiced design thinking and learned how to prioritize ideas through a method called “dot democracy,” in which each group voted on the best ideas before presenting them to the class.

The projects were further structured as posters for the final presentation on November 28^th^.

**November 13**^th^**:** Conversation circle with the President of Volunteering at HIAE, Ms. Telma Sobolh, and Introduction to Scientific Integrity with Ms. Deyse Noronha

The Volunteering team at HIAE has a history spanning more than 60 years, and its President, Ms. Telma Sobolh, spoke about the activities developed in Paraisópolis through the “Einstein Program in the Paraisópolis Community” (PECP - *Programa Einstein no Comunidade Paraisópolis*), which has been running for nearly 25 years. The program offers more than 30 courses. She also announced the opening of a new five-story building dedicated to an Integrated High School with Technical Education (ETIM - *Ensino Técnico Integrado ao Médio*) for the Paraisópolis community. Through a selective process, students are encouraged to apply for the technical course in hospital administration while attending high school.

In the second part of the activity, Ms. Deyse, a former resident of the Paraisópolis community and an HIAE employee since 2015, spoke about the key concepts of scientific integrity, emphasizing the importance of consent forms in conducting clinical research. She then asked the students how their lives had changed since joining the project. The most common responses were: “I’ve improved my communication skills,” “I’m doing better in science,” “It changed my perspective and opened my eyes,” “I’m more sociable,” and “It helped me think about my future and the career I want to pursue.”

**November 14**^th^**:** Gene therapy with Prof. Ricardo Weinlich and postgraduate student Isabella

At the beginning of the class, some foundational concepts were reviewed, including “What is DNA?”, “Where is DNA found?”, and “What is a nucleotide?” Isabella then provided several practical examples of how gene therapy enhances the agricultural aspects of daily life.

As the lesson advanced, the students explored how gene defects could be corrected using a guide RNA paired with the Cas9 protein, which cuts specific DNA sequences to correct pathological mutations. This technology is known as clustered regularly interspaced short palindromic repeat (CRISPR)-associated protein 9 (Cas9) gene editing. Isabella explained the need for a delivery system to transport guide RNA and Cas9 into cells, such as nanoparticles, which the students had previously studied, or viral vectors. Notably, one student, Wesley, observed that this delivery platform resembled a “Trojan horse.” Isabella and Ricardo also discussed the diseases that could benefit from this technology, highlighting ongoing studies at Einstein that focused on sickle cell disease and epidermolysis bullosa.

**Figure 13:**
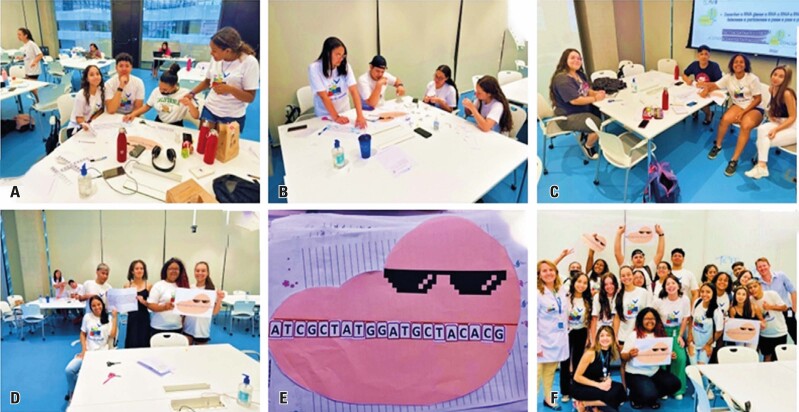
A to F: Class on gene therapy

In the second part of the class, the students participated in a hands-on activity in which they selected a guide RNA compatible with a specific DNA sequence, picking the appropriate nucleotides (adenine, thymine, cytosine, or guanine) and Cas9 protein (Figures 13A-F).

At the end of the class, Ricardo addressed the ethical questions surrounding gene therapy.

**November 21**^st^**:** Concepts of blood coagulation with Dr. João Guerra

On this day, the students learned about the key concepts of coagulation in the body and the diseases associated with its dysregulation, such as venous thrombosis and hemophilia. They also watched engaging videos and interacted with Dr. Guerra, the coordinator of the coagulation unit at the HIAE, to ask insightful questions during the presentations. It was gratifying to observe the students’ maturity at this stage. At the end of the activity, Dr. Guerra emphasized that “A good treatment [for coagulation disorders] depends on a good diagnosis.”

**November 22**^nd^**:** Poster training with Prof. Érika Rangel and postgraduate students Victoria and Guilherme Evangelista

Students practiced presenting their posters both as groups and individually. The postgraduate students posed scientific questions covering aspects such as study design, clinical implications, limitations, and future directions. This practice fostered self-confidence and improved the students’ communication skills, thus demonstrating student engagement.

**November 27**^th^**:** Academic trajectory with Prof. Juliana Magdalon and Blaidi Roberto Galvão, the High School and Technical Education Manager

On this day, Prof. Juliana asked the students about their future plans, including those beyond high school, and listened to the challenges they anticipated. The students then learned about the ETIM program in Healthcare Administration at the HIAE. The course will be offered free of charge in 2023 to residents of the Paraisópolis region and will be coordinated by the *Einstein Program in the Paraisópolis Community* (PECP). Blaidi explained the selection process, the number of available slots (40), the free tuition, and the program’s three-year duration. He also highlighted that classes in the course will be held full-time.

Next, the students heard from two graduates of the ETIM program in Nursing. These alumni successfully participated in the selection process of entering the *Einstein Nursing Faculty* and shared how they received tuition discounts while working as student assistants in the intensive care unit and in oncogenetics for four hours per day.

The students also attended talks with professionals representing various courses in the Faculty of Einstein. Prof. Welbert Pereira, the coordinator of the Biomedical Engineering program, introduced them to robotics, cell engineering, and artificial intelligence. They also learned about Einstein’s administration program from Prof. João Paulo Nascimento da Silva, who provided essential information on economic market trends and highlighted the partnership between Einstein and 30 public health units. The students’ learning was assessed using *Kahoot!* The winner Thiago Rodrigues Xavier de Oliveira won a book on administration.

Next, Prof. Angélica Cruz spoke about the physiotherapy program at Einstein, emphasizing the role of physiotherapy in disease and injury prevention and rehabilitation. She detailed various areas of specialization, including intensive care, cardiopulmonary care, oncology, teleconsulting, neurology, women’s health, and sports. The students also heard from Dr. Elda Maria Stafuzzo Gonçalves Pires, academic coordinator of the Medicine Program at FICSAE, who discussed the curriculum with a special focus on pediatrics and her field of expertise.

As a key goal, the students were encouraged to engage in the ETIM selection process. They were also motivated to plan their future with short-, medium-, and long-term goals.

**Figure 14:**
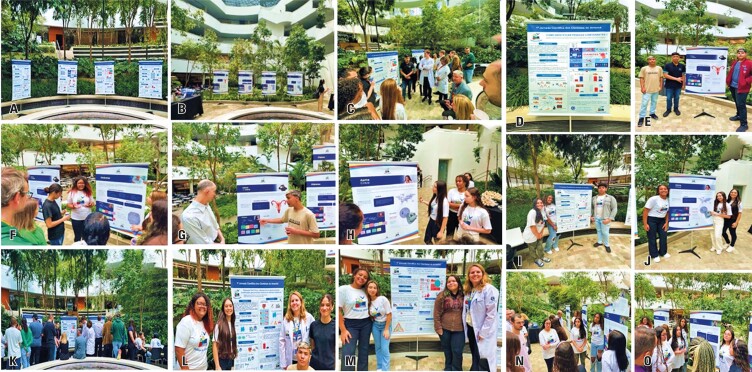
A to O: First scientific day with the Scientists of Tomorrow/*Cientistas do Amanhã*, held on November 28, 2023 at FICSAE

**November 28**^th^**:** On this day, the “First Scientific Day of the Scientists of Tomorrow” event showcased poster presentations from the design thinking activities focused on DM conducted with Prof. Érika Rangel on October 24^th^, along with the Innovation Team’s activities from November 7^th^ (Figures 14A-O). These were displayed as posters on the final day of the Scientists of Tomorrow/*Cientistas do Amanhã* project

Below are the poster titles and the authors, listed alphabetically:

#### a) *Diabetes mellitus*

Pre-clinical research on genetically modified stem cells to produce insulin in animals: Mariana Silva Barros Correia, Pablo Santos da Silva, Sophia Santos Dias da Silva, and Vitória Bezerra da Costa.

How can diabetes and pre-diabetes be prevented in children? Agatha Santos Almeida, Isamara de Barros Pereira, Jamilly Lima de Araújo, and Thiago Rodrigues Xavier de Oliveira.

How to identify people with diabetes? Kemily Vitória Novais Silveira, Vitória Santos, Weslley Batista Costa, and Yasmin Silva Cassimiro.

Treatment of pre-diabetes and diabetes to reduce cardiopathy: Alessandra Sousa Belarmino, Evellyn Luana Sampaio Correia, Jamiris Souza Neves, and Thomas Henrique Alves Oliveira.

#### b) Innovation

Innovation in asthma: Jamilly Lima de Araújo, Vitória Bezerra da Costa, and Vitória Santos.

Innovation in diabetes: Agatha Santos Almeida, Brena Rodrigues Felizardo, Isamara de Barros Pereira, Mariana Silva Barros Correia, and Sophia Santos Dias da Silva.

Innovation in ovarian cancer: Pablo Santos da Silva, Thiago Rodrigues Xavier de Oliveira, and Weslley Batista Costa.

Innovation in Duchenne muscular dystrophy: Alessandra Sousa Belarmino, Evellyn Luane Sampaio Correia, Jamiris Souza Neves, Kemily Vitória Novais Silveira, Thomas Henrique Alves Oliveira, and Yasmin Silva Cassimiro.

**Figure 15:**
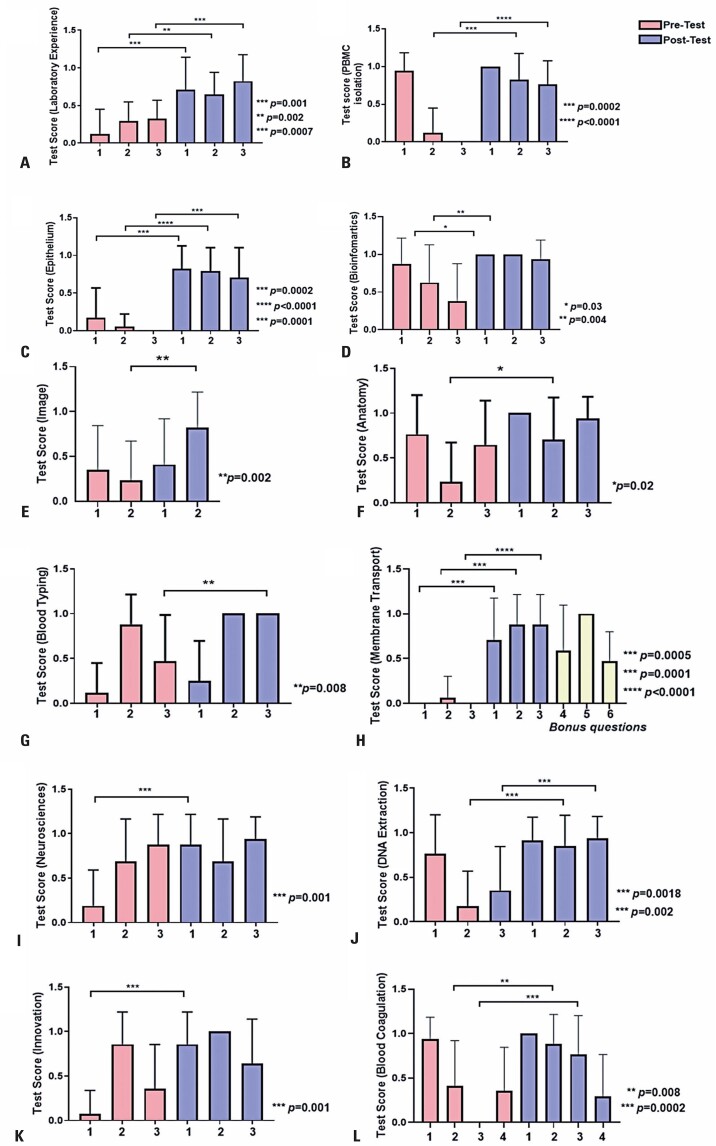
A to L: Results of the pre- and post-tests across the 12 activities conducted throughout the program

In this second edition of the Scientists of Tomorrow/*Cientistas do Amanhã* project, we also conducted statistical analyses using pre- and post-tests across 12 activities during the immersive training. Both multiple-choice and discursive questions were included. Across these analyses, we observed an increase in learning, although the extent of improvement varied both within and between activities (Figures 15A-L). Owing to non-normality in the data, we used the Wilcoxon matched-pairs signed-rank test with a significance threshold set at p<0.05.

In Activity 15H, which focused on the membrane transport of water using various concentrations of sodium chloride, we also included three bonus questions. These questions, which were not present in the pre-test, were added to the post-test to assess knowledge specifically related to the theoretical-practical content covered in the class.

**Figure 16:**
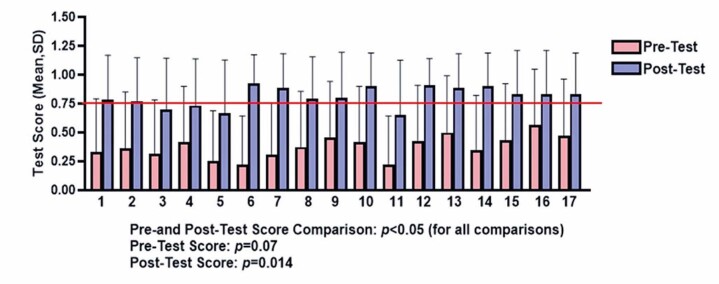
Pre- and post-test results of the 17 students. The test scores shown represent the mean and standard deviation (SD)

Notably, when applying the Kruskal-Wallis test to the pre-test scores among the 17 students, no significant difference was observed (p=0.07). However, post-test analysis revealed a significant difference (p=0.014), indicating heterogeneity in learning outcomes among students. However, in individual analyses using the Wilcoxon matched-pairs signed-rank test, all students demonstrated significant improvements (p<0.05). Furthermore, 13 of the 17 students (76.5%) achieved post-test scores exceeding 75% (Figure 16).

## DISCUSSION

Our study aimed to foster scientific thinking among elementary school students, with an emphasis on understanding scientific methodology through research-oriented theoretical and practical activities. We believe that our pedagogical approach will contribute to combating misinformation, which is crucial for building a more informed and critically engaged society.

In the second edition of the Scientists of Tomorrow/*Cientistas do Amanhã* project, we implemented strategic adjustments to create a more immersive and practical learning experience compared to the first edition. Our toolkit included various resources, such as pre- and post-tests^5^ with individual and collective statistical analyses throughout the project to measure knowledge and interactive competition using platforms such as the game-based platform *Kahoot!*,^6^ design thinking exercises,^7^ and poster presentations showcasing potential scientific inquiries.^8^

The pre- and post-tests enabled us to assess the students’ learning, both individually and collectively. All students displayed similar knowledge levels before class and showed improved test scores following the theoretical-practical activity. However, their post-test scores varied, indicating that some students performed better than others. This result demonstrates that our approach has pedagogical value and can be beneficial in educational and training contexts.^5^ Notably, the benefits of post-testing can also be observed with a retention test conducted seven days later, showing that post-tests promote better retention than pre-tests and support knowledge transfer to previously untested questions.^9^

The use of digital applications in education, such as the game-based platform *Kahoot!*, increased five-fold during the COVID-19 pandemic. A review of 12 studies on histology, anatomy, and medical education published between 2013 and 2021 indicated a generally positive student response, highlighting various benefits, such as collaborative learning, improved content knowledge, and increased attendance and participation.^10^ However, challenges remain, including the lack of control groups in studies and some negative aspects, such as increased time spent on tasks, cognitive overload from learning theory, and distractions from devices. A Brazilian study conducted between 2022 and 2023 compared a group using *Kahoot!* in histology classes, a group without any platform, and a group using a histological teaching platform.^6^ While the *Kahoot!* group performed better on the practical tests, the average scores showed no significant differences across the groups. Therefore, the game-based platform *Kahoot!* can be effectively combined with other teaching methodologies to create a dynamic learning environment and support student learning.

Design thinking is a creative, human-centered approach that fosters problem-solving skills essential for addressing complex, real-world challenges in an equitable, technologically feasible, and sustainable manner.^7^ This approach cultivates lifelong transferable skills, such as managing uncertainty and collaborative teamwork.^11^Following the two design thinking activities throughout the program—the DM activity with Prof. Érika Rangel on October 24^th^ and the Innovation Team session on November 7^th^—we developed posters for future presentations. These posters were thoughtfully structured, including an introduction, a clearly defined scientific question, the methodology for investigation, and the expected outcomes. These presentations offered a platform for students to share their research with peers, researchers, and other health professionals at the FICSAE, fostering dialogue and collaboration within the scientific community and enhancing the students’ communication skills, as this activity created opportunities for the students to build their self-confidence and helped them develop their academic voice.^12^

In this second edition of the project, three topics of scientific and practical relevance were introduced. One of these was an introduction to SUS, established in 1990.^13^ The SUS operates on three core principles: equity, universality, and integrality. These principles play critical roles in the production of medicines, vaccines, medical equipment, and immunobiology. Additionally, the SUS promotes public health initiatives, epidemiological surveillance, and workforce organization; supports basic sanitation; advances scientific, technological, and innovation efforts; enforces food and beverage quality standards; contributes to environmental preservation; and manages toxic and radioactive materials. However, despite the success of the SUS, challenges remain, including geographical disparities, limited funding, and suboptimal collaboration between the public and private sectors.

The second topic introduced was BLS training, an essential skill that should be learned early in life because of its critical role in first aid and life-saving care. Teaching BLS to elementary school students (mean age = 11.3 years) has proven to be effective, particularly when teachers receive prior training from specialized professionals.^14^ Moreover, studies show that 12-year-olds can perform CPR, including BLS and AED use, at levels comparable to those of adults,^15^ underscoring the importance of encouraging such training. In Brazilian schools, elementary school students have also demonstrated significant BLS learning gains, as assessed through pre- and post-tests.^16^

The third topic introduced genetic editing using CRISPR/Cas9 technology, focusing on the types of gene editing, delivery vectors, and disease characteristics.^17^ This allowed students to connect their prior knowledge of cell and tissue biology with cutting-edge technology and discuss the ethical issues researchers face today.

We have also placed significant emphasis on the development of soft skills. On the first day of the activities, we guided the students to form four groups, allowing them to choose peers with whom they felt comfortable and shared camaraderie. Later, we intentionally reorganized the groups to encourage interactions between students who may not have been previously connected to the school environment. We explained the importance of learning how to interact with others in life, even without prior affinity, as this provides opportunities to learn from each other, develop new skills, and gain deeper self-awareness.

Throughout all activities, Prof. Érika Rangel, a physician-researcher at Einstein, actively engaged with the students to encourage participation, help them with their project topics, and foster an informal atmosphere where students felt comfortable asking questions, seeking clarification, and sharing experiences. She progressively encouraged the students to present their activities orally and in writing, enhancing their communication skills. Over time, most students became more confident in expressing their opinions, including speaking with a microphone—an action that truly “gave voice” to the students.

Although the hawthorne effect—a phenomenon in behavioral and social research where individuals change their behavior simply because they know they are being observed or studied—may be present, the strategies adopted in the Scientists of Tomorrow/*Cientistas do Amanhã* project have ensured fruitful learning for students throughout their journey. The Hawthorne Effect can influence the outcomes of randomized controlled trials across various fields, including diabetes technology,^18^ chronic rhinosinusitis treatment,^19^ and psychiatric disorders.^20^ This effect, driven by participants’ psychological and behavioral responses, can cause placebo groups to perform similarly to the treated groups, even in terms of biochemical parameters^21^, emphasizing the importance of control groups in research. Conversely, the Hawthorne Effect has been shown to positively affect hand hygiene compliance in an intensive care unit, reducing infection rates by 23%.^22^ Furthermore, standardizing methodologies for measuring the Hawthorne Effect is essential for critically evaluating clinical study results.^23^

The Scientists of Tomorrow/*Cientistas do Amanhã* project aligns with the SDGs set by the United Nations in 2015, which serve as a universal call to action to end poverty, protect the planet, and ensure that all people can enjoy peace and prosperity by 2030. Each action of the 17 integrated SDGs impacted the others, reinforcing a holistic approach. Specifically, this project contributes to SDG 4, promoting quality education,^24^ and SDG 10, focusing on reducing inequality.^25^SDG 4 emphasizes “inclusive and equitable quality education and lifelong learning opportunities for all.” Through our project, we support this by enhancing skills among youth, particularly those in vulnerable situations, equipping them with technical and vocational competencies for employment, decent work, and entrepreneurship. In this project, the students also received training that supported their enrolment in higher education, including vocational training, information and communications technology, and technical and scientific programs, with both boys and girls gaining literacy and numeracy skills.

SDG 10 (“Reduced Inequalities”) aims to “empower and promote social inclusion,” “ensure equal opportunities and reduce inequalities of outcomes,” and “ensure enhanced representation and voice.” Our project contributes to fostering these values and promoting equitable access to educational and career opportunities.

The project’s mission also resonates with the Jewish concept of Tikkun Olam, or “repairing the world,” which advocates actions aimed at improving society and the environment.

To evaluate the impact of the Scientists of Tomorrow/*Cientistas do Amanhã* project, we will track the students’ progress over a 10-year period, assessing the number of students advancing to high school, enrolling in technical schools, or entering higher education and the job market. With a growing number of participants, we anticipate developing a comparison indicator between students from the same school who participated in the project and those who did not, thus providing further insight into the project’s long-term benefits.

Our study has some limitations, including possible student selection bias, the lack of a control group to measure the Hawthorne Effect, and the small number of students from a single school.

## CONCLUSION

The Scientists of Tomorrow/*Cientistas do Amanhã* project is an innovative and reproducible project that allows elementary school students to skillfully apply the knowledge needed for efficient and effective actions that integrate scientific and technological support and values. These will enable them to seek continuous improvement and self-development through studies and research; identify and critically incorporate new methods, techniques, and technologies into their practices; and respond to daily and unexpected situations with flexibility, resilience, and creativity.
